# Optimizing the Extraction and Encapsulation of Mucilage from *Brasenia Schreberi*

**DOI:** 10.3390/polym11050822

**Published:** 2019-05-07

**Authors:** Qingying Luo, Min Wu, Yanan Sun, Junxia Lv, Yu Zhang, Hongfu Cao, Dingtao Wu, Derong Lin, Qing Zhang, Yuntao Liu, Wen Qin, Hong Chen

**Affiliations:** College of Food Science, Sichuan Agricultural University, Yaan 625014, Sichuan, China; cherry12112009@163.com (Q.L.); xiaotaiyangwumin@163.com (M.W.); sicausunyanan@163.com (Y.S.); 15709482720@163.com (J.L.); zyzhangyu1205@163.com (Y.Z.); 18728191838@163.com (H.C.); DT_Wu@sicau.edu.cn (D.W.); lindr2018@sicau.edu.cn (D.L.); zhangqing@sicau.edu.cn (Q.Z.); lyt_taotao@163.com (Y.L.); qinwen@sicau.edu.cn (W.Q.)

**Keywords:** *Brasenia schreberi*, mucilage, high-speed shear-assisted extraction, optimal extraction, capsule

## Abstract

The mucilage from *Brasenia schreberi* (BS) exhibits various biological activities, including antialgal, antibacterial, soluble-fiber properties, and excellent lubricating behavior. Thus, the extraction and wide use of mucilage in the food industry are crucial. In this study, the high-speed shear-assisted extraction of mucilage from BS was optimized by using response surface methodology (RSM). The optimal extraction conditions were as follows: Extraction temperature of 82 °C, extraction time of 113 min, liquid–solid ratio of 47 mL/g, and shear speed of 10,000 rpm. Under these conditions, the actual yield of BS mucilage was 71.67%, which highly matched the yield (73.44%) predicted by the regression model. Then, the BS mucilage extract was powdered to prepare the capsule, and the excipients of the capsule were screened using a single-factor test to improve the disintegration property and flowability. The final capsule formulation, which consisted of: 39% BS mucilage powder (60 meshes); 50% microcrystalline cellulose (60 meshes) as the filler; both 10% sodium starch glycolate and PVPP XL-10 (3:1, 60 meshes) as the disintegrant; both 1% colloidal silicon dioxide and sodium stearyl fumarate (1:1, 100 meshes) as the glidant by weight; were used for preparing the weights of a 320 mg/grain of capsule with 154.7 ± 0.95 mg/g polysaccharide content. Overall, the optimized extraction process had a high extraction rate for BS mucilage and the capsule formulation was designed reasonably.

## 1. Introduction

The perennial water plant *Brasenia schreberi* (BS) is an invasive aquatic weed found in the USA but is considered a kind of medicinal and edible plant in Asia, where it is cultivated and traded [1]. In China, BS has been consumed as a functional food for millennia owing to its high nutritional value and biological activities, including antibacterial, antioxidant, anti-inflammatory, hypolipidemic, and cholesterol-lowering effects [2,3,4]. The submersed organs of BS, including the young stems, buds, and the undersides of young leaves, are all covered by a thick and clear mucilaginous substance [5]. The mucilage in BS is endowed with the high amounts of various nutrients and has elicited much interest in recent years because of their biological activities, such as antibacterial, antialgal, and soluble-fiber properties, and excellent lubricating behavior [6,7,8]. Thus, the extraction and wide use of mucilage in the food industry are crucial. Recently, Zhao [9] employed ultrasonic treatment to extract mucilage from *Arabidopsis* with high efficiency and simple procedure. However, the resulting extraction buffer was limited because it became turbid and could not be readily filtered. Nazir [10] and Miart [11] utilized MuSeeQ for aqueous extraction of basil mucilage, however, this method is time-consuming. No specific report regarding the extraction of mucilage from BS has been published. Moreover, BS mucilage products are limited in the manufacturing of canned fresh rough-wrought products and results in low efficiency. Novel methods for deep-processing BS mucilage with high added value have urgent significance.

Capsules are tasteless and odorless delivery systems for powdered medicine and have been used since the late 19th century [12,13]. Capsulation is defined as a solid preparation made by filling drugs or supplementary materials in hollow-hard or elastic-soft capsules, that can release their contents at controlled rates under specific conditions. The choice of materials and the methodology for capsulation are dependent on the active agent in question and the target application [14]. Over the last few years, the absorption of therapeutic capsules has received great interest because of their high bioavailability, fast absorption and disintegration, good stability, simpler production process compared with other administration routes, and ability to offset the bitterness and odor of materials [15,16]. A large number of bioactive substances, such as polysaccharides, polyphenol, flavors, and fragrances, are extensively used in the preparation of capsules owing to the rapid developments in capsule preparation and expansion of production [17,18,19].

In this study, mucilage was extracted from BS through a high-speed shear-assisted method because of the abundant human health-promoting effects of mucilage and distinguished advantages of capsule preparation. The extraction conditions were optimized, and the resulted mucilage extract was further developed into capsules. The flowability of the resulting BS mucilage powder was assessed on the basis of the angle of repose, bulk density, and tapped density. Then, the capsular formulation was determined. The excipient formulation of the capsule was screened, and the capsule, along with polysaccharide, weight, and water content, was evaluated, thereby providing basic pharmaceutical research for the preparation of capsules by BS mucilage.

## 2. Materials and Methods

### 2.1. Materials and Reagents

The fresh *Brasenia schreberi* was harvested in summer in Shizhu County (Chongqing, China). Glucose standard substance (AR) was purchased from Chengdu Kelong Chemical Reagent Factory (Sichuan, China). The anthracenone (AR) was purchased from Shanghai Kefeng Industrial Co. Ltd. (Sahanghai, China). The crospovidone (PVPP XL and PVPP XL-10) were both purchased from International specialty company ISP, Wayne, NJ, USA. Croscarmellose sodium (CCS) was purchased from Changwei Pharmaceutical Co., LTD (Shanghai, China). The pregelatine starch, water-soluble starch, microcrystalline cellulose, calcium hydrophosphate, colloidal silicon dioxide and sodium stearyl fumarate were all purchased from Shanhe Co., LTD (Anhui, China). The magnesium stearate was purchased from Yunhong Chemical Preparation Accessories Technology co., LTD (Shanghai, China). The sodium carboxymethyl starch (CMS-Na) and microcrystalline cellulose were purchased from Germany JRS, Shanghai, China. The cellactose (80 meshes) was purchased from Germany Merlot, Shanghai, China. The sulfuric acid, ethanol and other reagents were all used as analytical purity.

### 2.2. Extraction Optimization of BS Mucilage

#### 2.2.1. Extraction of BS Mucilage

The fresh BS was pretreated by flushing with water for 5 min. Briefly, BS mucilage was extracted by high-speed shearing with 0.3 mol/L sodium hydroxide according to the designed liquid–solid ratio, extraction temperature, extraction time and shear speed. The supernatant was filtered, followed by centrifugation at 6000 rpm for 30 min. Next, the filtrate was concentrated to a 1/3 volume of the primary volume using a rotary evaporator under reduced pressure, and then the mucilage was obtained by drum wind drying oven at 60 °C.

Extraction yield of BS mucilage (%) = mass of extracted mucilage after drying (g)/mass of BS taken for extraction (g) × 100%.

#### 2.2.2. Experimental Design for Response Surface Methodology (RSM)

The effects of the four independent processing parameters: The extraction temperature (A, °C), liquid–solid ratio (B, mL/g), extraction time (C, min) and shear speed (D, rpm), on the dependent variables were investigated using RSM. The central composite design (CCD) for RSM required five levels, coded as −2, −1, 0, +1, and +2. As shown in Table 1, the total number of experiments designed was 26 based on the five levels and a four-factor experimental design, with five replicates at the central conditions of the design for estimation of a pure error sum of squares. The dependent variable was the extraction yield of BS mucilage (*Y*). The model equation for the response (*Y*) to the three independent variables (A, B, C and D) is given in the Equation (1):

(1) $$Y=\beta_{0}+\sum_{i=1}^{2}\beta_{i}X_{i}+\sum_{i=1}^{2}\beta_{ii}X_{i}^{2}+\sum_{i}\sum_{j=i+1}\beta_{ij}X_{i}X_{j}$$

where *X_i_* is the encoded independent variable affecting the response *Y*; *β*_0_, *β_i_* (*i* = 1, 2, …, *k*), *β_ii_* (*i* = 1, 2, …, *k*) and *β_ij_* (*j* = 1, 2, …, *k*) are the regression coefficients for intercept, linear, quadratic, and interaction terms, respectively; *k* = 3; *i* < *j*.

### 2.3. Preformulation Study

#### 2.3.1. Influence Factor Test

The dried BS mucilage extract was smashed and screened through 60 meshes. Then, the extract and resulted powder were both inspected the appearance visually, and the water content of the powder was measured by the hypobaric drying method, according to the People’s Republic of China Pharmacopeia. The powder was placed in a temperature of 60 °C, a relative humidity of 95% ± 5%, and illuminance of 4500 lx ± 500 lx for consecutive 10 days, respectively. Subsequently, the appearance, weight and polysaccharide content of the powder were determined on the 5th and 10th days.

#### 2.3.2. Angle of Repose

Prior to determining capsular formulation, the flowability of BS mucilage powder was assessed by determining several parameters, including the angle of repose, bulk and tapped densities. To achieve an accurate result, each test was repeated three times and the average was taken. The angle of repose was determined according to the funnel method, and the mean diameter of the base of the powder mixture cone and the tangent of the angle of repose were determined using Equation (2) [20].

θ = tan^−1^(*h*/*r*) (2)

where θ is the angle of repose, *h* is the cone height, and *r* is the cone base radius.

#### 2.3.3. Bulk Density and Tapped Density

To measure density, the granules were filled in a 100 mL capacity measuring cylinder up to at least 3/4 the height (Bulk Density Apparatus, Shivani Scientific Inds., Mumbai, India). The bulk density is the quotient of weight to the volume of the sample. The tapped density is the quotient of the weight of the sample to the volume after taping a measuring cylinder 500 times from a height of 1.5 in Reference [21].

### 2.4. Preparation of the Capsule with BS Mucilage

The pregelatinized starch, water-soluble starch, microcrystalline cellulose (MCC), calcium hydrogen phosphate, lactose, sodium starch glycolate (CMS-Na), croscarmellose sodium (CCS), crospovidone (PVPP XL and PVPP XL-10), colloidal silicon dioxide, magnesium stearate and sodium stearyl fumarate were used as excipients to screen formulation of the capsule by a single-factor assay, on the basis of the disintegration time as determined by disintegration apparatus (LB-812, Yellow-Sea testing instrument factory, Shanghai, China) and the angle of repose, according to the Chinese Pharmacopeia. The proposed formulation consisted of a uniform mixture of 124.8 mg of BS mucilage powder, 160 mg of filler, 32 mg of disintegrant, 3.2 mg of glidant to prepare a 320 mg/granule capsule.

### 2.5. Identification by Thin-Layer Chromatography

An amount of 2 g of the BS mucilage was dissolved in 100 mL of 70% methanol. The sample was taken to the ultrasound for 30 min for complete solubilization. The thin-layer chromatography (TLC) plates coated with the silica gel (100 m × 200 m) were supplied by Marine chemical plant (Qingdao, China). The two-dimensional thin layer chromatogram was developed using: dichloromethane; methanol; water (8:1:0.1) and ethyl acetate; methanol; water (8:1:0.5) as the mobile phase solvents. The plates were dried before spraying 3% aluminum trichloride (spraying agent) and visualized under a long UV light at 360 nm to detect the presence of polysaccharides.

### 2.6. Determination of Polysaccharide Content

#### 2.6.1. Preparation of Glucose Standard Solution

The glucose standard substance (25.7 mg) was precisely weighed using METTLER AE240 electronic weighing balance (Mettler Toledo, Zürich, Switzerland) and dissolved in 5 mL of 0.1 mol/L sodium hydroxide solution. The pH value of the mixture was adjusted to 6.8–7.0 with 0.1 mol/L diluted hydrochloric acid then transferred into 50 mL volumetric flask followed by mixing of the mixture.

#### 2.6.2. Standard Curve Manufacture

Different volumes of glucose standard solution (0, 0.5, 1, 1.5, 2, 2.5, and 3 mL) were pipetted respectively into seven 25 mL volumetric flasks and shaken up after a slow addition of 6 mL of 0.2% anthranone-sulfuric acid solution. The mixture was bathed for 8 min in boiling water and then cooled to room temperature. A solvent-free sample was set as a reference and made zero. Scanning in the wavelength range of 200–700 nm by ultraviolet spectrophotometer spectrophotography (Shanghai Youke Instrument Co., LTD, Shanghai, China), and 625 nm was determined as the best detection wavelength. The record keeping of the absorbance values at 625 nm and the glucose standard curve was done using Excel. The regression equation of the standard curve was obtained as *Y* = 17.39*X* + 0.00913, *R*^2^ = 0.9998.

### 2.7. Statistical Analysis

All experiments were performed at least three times. The analyses of all samples were run in triplicate and averaged. All values were presented as the mean ± standard deviation (SD). The statistical analysis was performed using a one-way ANOVA in SPSS 17.0 for Windows (SPSS Inc., Chicago, IL, USA). Duncan post hoc tests were performed when significant differences were found. *P* < 0.05 was considered to be significant and *P* < 0.01 was regarded as highly significant.

## 3. Results and Discussion

### 3.1. Optimization of Extraction Conditions

Response surface methodology (RSM) is a statistical technique usually used for quantitative data from suitable experimental designs and for determining and solving multivariate equations. The application of RSM in the optimization process may save cost, time, and energy [22]. The central composite design is widely used for fitting a second-order method in RSM, which consisted of four runs at the corners of the square, four runs at the center of this square, and four axial runs. It is also used for estimating the parameters of a full second-order polynomial model [23]. In the present study, the results of 26 trial points tested in a random order based on the CCD by Design Expert software of version 8.0.6.1 (Stat-Ease Inc., Minneapolis, MN, USA), including the design and experimental values, are presented in Table 1. The experimental data obtained from the multiple regression analysis method showed that the predicted response *Y* for the extraction yield of BS mucilage could be fitted into the following second-order polynomial equation, as shown in Equation (3):

*Y* = 68.95 + 1.83A + 4.70B + 1.96C + 2.13D + 0.21AB − 1.34AC + 0.097AD + 2.56BC + 0.85BD + 2.17CD − 3.21A^2^ − 5.36B^2^ − 4.44C^2^ − 1.00D^2^ (3)

where *Y* is the extraction yield of BS mucilage (%); and A, B, C, and D are the coded values of the tested extraction temperature, liquid–solid ratio, extraction time, and shear speed, respectively.

The significance of the obtained experimental data was evaluated, and the adequacy and fitness of the model were analyzed with analysis of variance (ANOVA) [24,25]. According to the results in Table 2, the high *F* value (13.09) and low *P*-value (*P* < 0.0001) suggested the significance of the regression models. The lack of fit was nonsignificant (*F* value = 17.76, *P*-value = 0.1828 > 0.05), indicating that the model was adequate for predicting the yield of BS mucilage. The high coefficient (*R*^2^ = 0.9434) and the adjusted determination coefficient (*R*^2^_Adj_ = 0.8713) indicated a high correlation between the predicted and experimental values. Furthermore, the *P*-values were used for checking the significance of each coefficient, and a large F value and small *P*-value corresponded to high significance [26,27]. As a result, the linear (A, B, C, and D), interaction (BC and CD), and quadratic coefficients (A^2^, B^2^, and C^2^) were significant (*P* < 0.05), whereas, the other coefficients (AB, AC, AD, BD and D^2^) were nonsignificant (*P* > 0.05).

**Figure 1:**
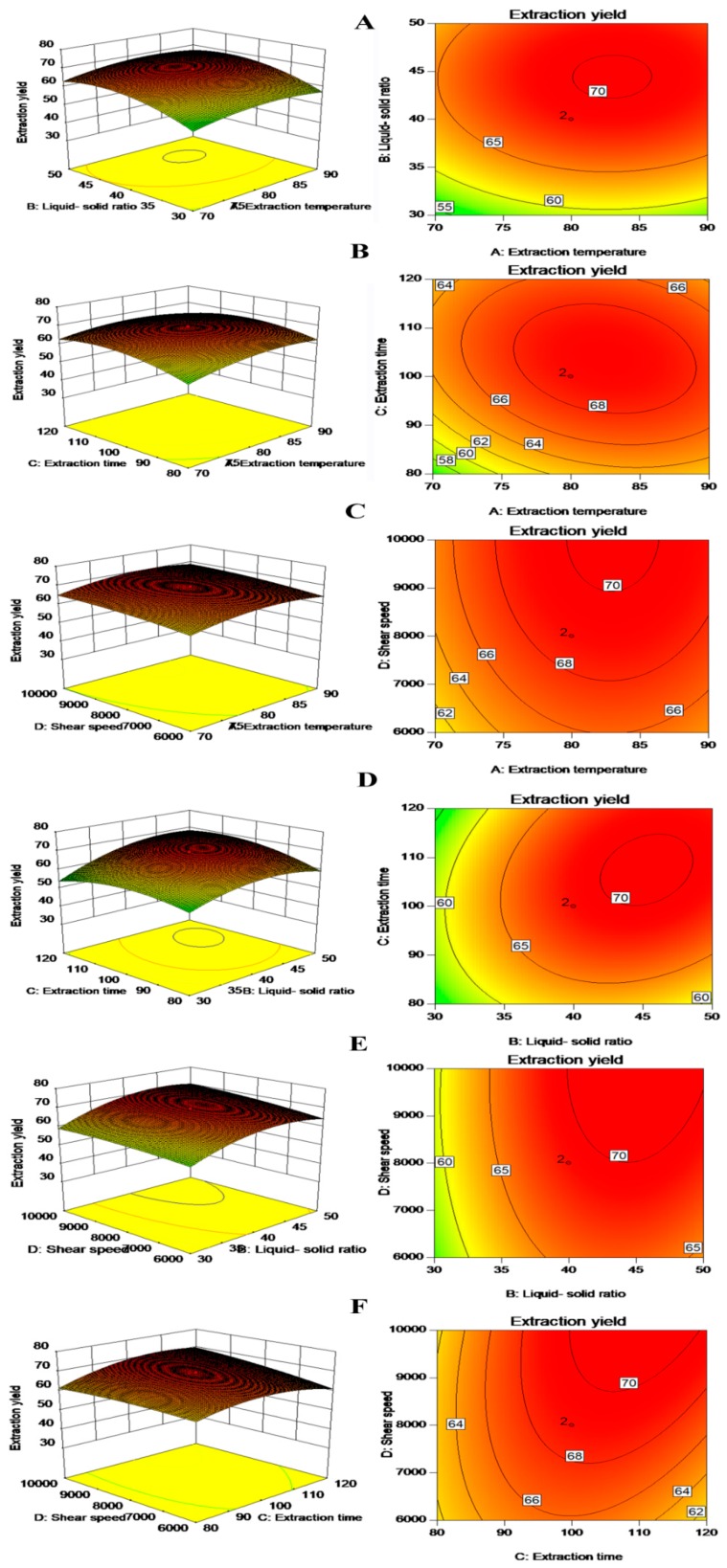
The 3D response surface and 2D contour plots showing the effects of extraction factors on the yield of BS mucilage. (**A**) 3D response surface and 2D contour plots showing the effects of liquid–solid ratio and extraction temperature on the yield of BS mucilage; (**B**) 3D response surface and 2D contour plots showing the effects of extraction time and extraction temperature on the yield of BS mucilage; (**C**) 3D response surface and 2D contour plots showing the effects of shear speed and extraction temperature on the yield of BS mucilage. (**D**) 3D response surface and 2D contour plots showing the effects of extraction time and liquid–solid ration on the yield of BS mucilage. (**E**) 3D response surface and 2D contour plots showing the effects of shear speed and liquid–solid ratio on the yield of BS mucilage. (**F**) 3D response surface and 2D contour plots showing the effects of shear speed and extraction time on the yield of BS mucilage.

Three-dimensional (3D) response surface and two-dimensional (2D) contour plots were provided as graphical representations of the regression equation (Figure 1). The interaction effects between the variables were exhibited by the shapes of the contour plots. An elliptical contour plot indicates a significant interaction among corresponding variables, whereas, a circular contour plot indicates a nonsignificant interaction among corresponding variables [28]. Figure 1A shows the effects of the liquid–solid ratio and extraction temperature on the extraction yield of the BS mucilage. The BS mucilage yield increased when the liquid–solid ratio increased in the range of 35–55 mL/g and extraction temperature increased in the range of 70–95 °C, the yield then decreased after 55 mL/g and 95 °C. Figure 1D shows the effects of the extraction time and liquid–solid ratio on the yield of BS mucilage. The yield of BS mucilage increased with increasing extraction time and an increasing liquid–solid ratio, but the yield then decreased as these two variables increased further. The response surface slope of the interaction between the extraction time and liquid–solid ratio was steep and the contour plot was elliptical, which suggested that the interaction between the two variables was extremely significant [29]. Figure 1E evaluates the effect of the shear speed and liquid–solid ratio on the yield of BS mucilage. A maximum yield (approximately 69%) of BS mucilage was obtained when the liquid–solid ratio was set at approximately 40 mL/g and the shear speed at approximately 8000 rpm. However, the contour plot was circular and the slope was gentle, indicating that the mutual interaction between liquid–solid ratio and the shear speed was nonsignificant. From the analysis of the interactions between variables, two such interactions (the extraction time and liquid–solid ratio; the shear speed and extraction time) among the tested variables were significant (*P* <0.05). Among the four variables, the liquid–solid ratio was the most significant factor affecting the BS mucilage extraction yield, according to the *P*-value, and the gradient of the slope in the three-dimensional response surface map. The next most significant factor was the shear speed, followed by the extraction time, and lastly, was the extraction temperature (Table 2 and Figure 1).

The optimum extraction conditions for the maximum extraction yield of the BS mucilage, recommended by the Design-Expert software, were as follows: An extraction temperature of 81.92 °C, liquid–solid ratio of 46.73 mL/g, extraction time of 112.64 min, and shear speed of 9999.99 rpm. Validation experiments were carried out under these conditions with slight modifications: An extraction temperature of 82 °C, liquid–solid ratio of 47 mL/g, extraction time of 113 min, and shear speed of 10 000 rpm. Under these conditions, the actual yield of BS mucilage obtained was 71.67%, which highly matched the yield (73.44%) predicted by the regression model. These results indicated that the model was accurate and adequate in predicting the BS mucilage extraction conditions.

### 3.2. Evaluation of the BS Mucilage

**Figure 2:**
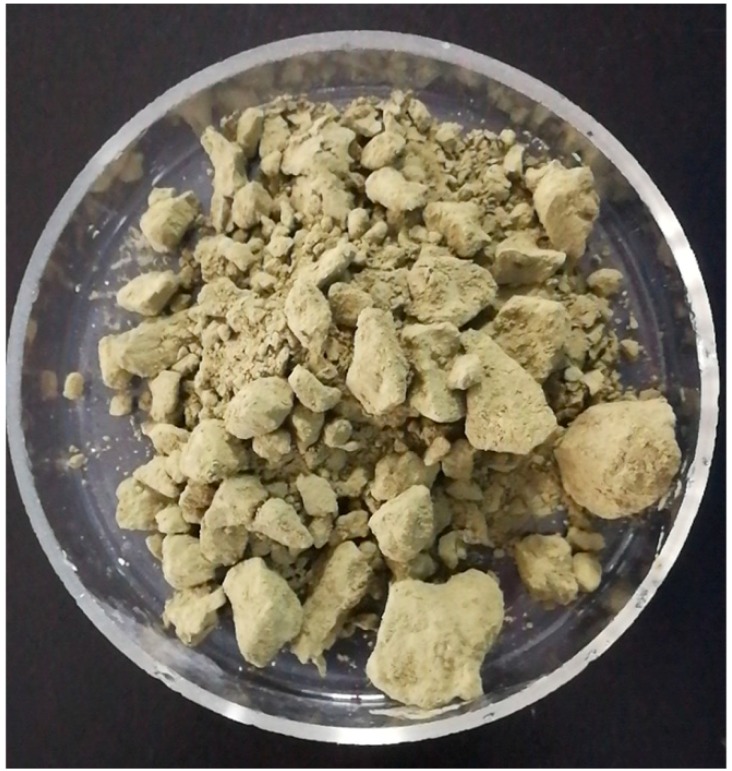
The image of the powder of BS mucilage extract.

After drying, the BS mucilage extract became a yellow-brown solid with a smooth surface (Figure 2). The mean value of water content was 7.17% ± 0.481% and the polysaccharide content was 53.49% ± 0.013%. The resulting BS mucilage powder was yellow, greasy, slippery, sweet, and astringent. The angle of repose has been defined as an indirect measure of particle size, shape, porosity, cohesion, fluidity, surface area, and bulk. It provides an indication of the ease at which the powder flows. A small angle of repose (less than 40°) indicates a free-flowing powder, whereas, large angles (more than 50°) indicate poor flow properties [30]. The mean angle of repose of BS mucilage was 38.91° ± 1.527°, indicating that the BS mucilage had good flowability. The bulk and tapped densities are common in process powder attributes tested during pharmaceutical product development and manufacturing, which predict the capacity of a powder to be compressed and reflect the importance of interparticulate interactions [31]. The mean bulk and tapped densities of the BS mucilage were 0.5 ± 0.017 g/mL and 0.59 ± 0.010 g/mL, respectively. In a free-flowing powder, the bulk and tapped densities would be close in value. Collectively, these results suggested that the granular formulation prepared using BS mucilage as a major constituent, possessed excellent flowability.

High-temperature, high-humidity, and intense light exposure tests were employed herein to further investigate the stability of the BS powder and provide basic data for the package and storage of the capsule by BS mucilage. The results showed obvious alterations in the appearance of BS mucilage under high temperature (60 °C), high humidity (95% ± 5%) and strong light (4500 lx ± 500 lx), whereas, the weight and polysaccharide contents did not change markedly (Table 3). For example, BS mucilage melted and coagulated at high temperatures and gradually darkened under high-humidity and strong light exposure. Consistently, these findings indicated that the BS mucilage should be kept in low-temperature, low-humidity, and away from light.

### 3.3. Preparation of Capsule by BS Mucilage

To maximize the use of the mucilage from BS, we further prepared it into capsules. Gelatin is a protein derived from denatured collagen that contains high levels of hydroxyproline, proline, and glycine. It is useful as a thermally reversible gelling agent for capsulation [32]. Given that the BS mucilage was unstable under high-temperature and humidity, and strong light exposure, purple gelatin was selected with a blister package as a capsule shell because of its excellent protection from light, membrane formation, biocompatibility, and nontoxicity.

During the commercial production of capsules, a blend with good flow characteristics is desirable for smooth operation and efficient production. If the blend has poor flow characteristics, then weight variation problems may arise. Ideally, the powders used for capsule filling should have a good flow property, that is, they should produce an even packing density (i.e., the particles of each component should have a particle size and densities as close as possible to avoid segregation, or de-mixing, and the powder should not be adhesive to the surface of either capsule bodies) [33]. Moreover, to ensure a drug is readily available for absorption as immediately as possible, one should focus on the formulation design of immediate-release oral capsules that can achieve rapid disintegration and dissolution. However, the formulation of certain active pharmaceutical ingredients cannot be achieved adequately with the use of a single-component excipient. Hence, excipients with inherent multifunctional characteristics, such as a better flow, minimum tendency for segregation, high compressibility, rapid disintegration ability, and low or no moisture sensitivity, have been extensively used in current pharmaceutical formulation development studies [34,35]. In our study, the results showed that microcrystalline cellulose (MCC), dicalcium phosphate, croscarmellose sodium (CCS), sodium starch glycolate (CMS-Na), crospovidone (PVPP XL or PVPP XL-10), colloidal silicon dioxide, magnesium stearate, and sodium stearyl fumarate were not absorbed at 625 nm by a UV spectrophotometer, and they were employed for the subsequent excipient screening.

Capsules must be disintegrated before the drug in the capsule is absorbed by the human body. The most widely used superdisintegrants are PVPP, CCS, and CMS-Na [36]. Herein, CCS, CMS-Na, PVPP XL, and PVPP XL-10 were measured by disintegration time with a disintegration apparatus for the screening of the best disintegrant of the capsule with the BS mucilage. Table 4 shows that the disintegration effect of CMS-Na was better than that of CCS and the disintegration effect of the mixture of CMS-Na and PVPP XL-10 were enhanced in a dose-dependent manner. Relatively small amounts of superdisintegrants, typically about 5–10% by weight, which is enough to influence the disintegration performance in the drug delivery systems through liquid transport enhancement by rapid swelling, as well as gel formation, are usually added to tablet formulations [37]. Herein, CMS-Na, and PVPP XL-10 (3:1, 60 meshes) were selected as the disintegrant, with an amount of 10% to prepare a capsule with the BS mucilage.

Disintegrants are important components of the capsule formulation to establish rapid release properties. However, other excipients may also have effects. MCC is a widely used flowability aid that can be pre-granulated or directly compacted with high hygroscopy [38]. In addition to the effect of disintegrants, MCC also enhances liquid transport into a tablet matrix, thereby accelerating diffusion and capillary action [39]. In Table 5, the results showed that the disintegration time and angle of repose in the MCC (JRS) group were lower compared with other groups, and the viscous layer of the MCC was more dispersed with better liquidity than that of calcium hydrophosphate. Calcium hydrophosphate is an insoluble, non-swelling, and high-density filler, which may have resulted in slower disintegration time. By contrast, MCC has a very high intra-particle porosity, which generally promotes swelling and disintegration of MCC-containing tablets by capillary action, which resulted in the short disintegration time [40]. As a result, MCC (60 meshes, JRS) was selected as the filler, with an amount of 50% to prepare the capsule with the BS mucilage.

Other excipients, such as sodium stearyl fumarate, magnesium stearate, and colloidal silicon dioxide, are commonly added to the formulation for the production of dosage forms with desirable capsule properties, in terms of the fill weight and flowability [38]. In Table 6, the angle of repose in the For-4 group (colloidal silicon dioxide:sodium stearyl fumarate = 1:1) was the lowest, which indicated that colloidal silicon dioxide and sodium stearyl fumarate used together could improve the liquidity best. Overall, a 39% powder of BS mucilage (60 meshes), 50% MCC (60 meshes, JRS) as filler, 10% CMS-Na and PVPP XL-10 (3:1, 60 meshes) as the disintegrant, 1% sodium stearyl fumarate and colloidal silicon dioxide (1:1, 100 meshes) as the glidant by weight, were used for the preparation of a 320 mg/grain capsule.

### 3.4. Evaluation of the Capsules

**Figure 3:**
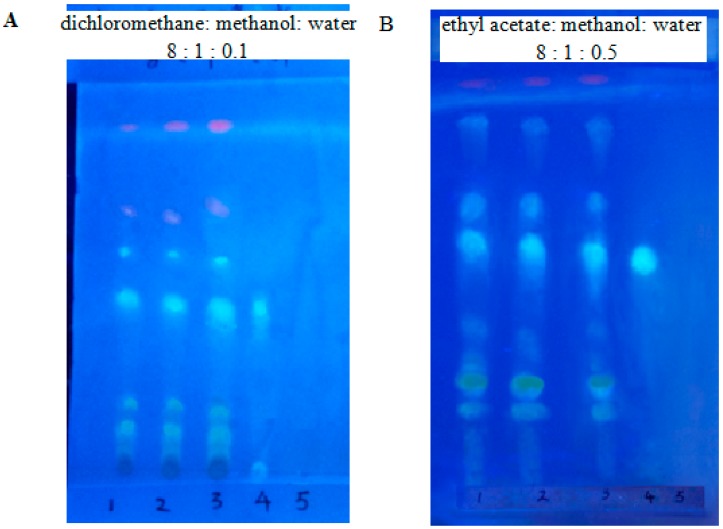
Isolation of polysaccharide from capsules made by BS mucilage by thin layer chromatography and its visualization under far UV light. (**A**) TLC image with dichloromethane:methanol:water = 8:1:0.1 as the developing solvent; (**B**) TLC image with ethyl acetate:methanol:water = 8:1:0.5 as the developing agent. 1–3, 4 and 5 refer to BS mucilage capsule samples, quercetin reference and excipient reference, respectively.

The capsule produced by BS mucilage was light-yellow or yellow and had a sweet flavor and slightly astringent aftertaste. As illustrated in Table 7, the water content in the different batches of capsule ranged from 4.56% to 5.22%, all less than the stipulated 9% according to the Chinese Pharmacopeia. The average weight of the capsule was in conformity with the range from 0.3261 g to 0.3282 g. The thin layer chromatographyis widely used for the analysis of several phytochemicals. The advantages of using TLC to identify the target component of herbal medicines are its simplicity, versatility, high velocity, specific sensitivity, and simple sample preparation [41]. Thus, TLC was performed for the identification of the polysaccharide from the capsule. Figure 3 reveals the presence of polysaccharides in the capsule made by BS mucilage, and then the content of polysaccharides as 154.7 ± 0.95 mg/g, which was measured by using the anthranone-sulfuric acid method with glucose as a standard at 625 nm.

The BS mucilage consists of a gelatinous polysaccharide coating composed of approximately 32–40% d-galactose, 19–29% d-glucuronic acid, 13–16% l-fucose, 10–14% D-mannose, and other monosaccharides. Mannose forms the backbone of the polymer and galactose forms its side chains [42]. Polysaccharides are one of the main components of BS mucilage and show promise for use in a wide range of functional food and pharmaceutical products. Recently, polysaccharides derived from natural resources have attracted extensive attention owing to their diverse types of biological activities, such as antioxidative, immunomodulatory, antidiabetic, antibiotic, antimicrobial, and anti-inflammatory properties [43]. In our study, to ensure the capsules made by BS mucilage containing polysaccharides are available for absorption as immediately as possible, the excipients that showed good flow characteristics were blended with BS mucilage to prepare the capsule. Considering the BS mucilage was unstable, purple gelatin was selected as a capsule shell to protect it from the outside environment and delay the decomposition. After the encapsulation of BS mucilage, the stability was better than the extract, based on their determination. Taken together, our results showed that the process of BS mucilage encapsulation was low-cost, efficient with regard to production, and convenient in operation, which is suitable for large-scale commercial production. Future studies must explore the BS mucilage capsules by pharmacokinetic evaluation, dissolution testing in vitro and the pharmacological effects in animal models.

## 4. Conclusions

In the present work, the optimal conditions of high-speed shear-assisted extraction optimized by RSM were as follows: An extraction temperature of 82 °C, liquid–solid ratio of 47 mL/g, extraction time of 113 min, and shear speed of 10,000 rpm, with the actual yield of BS mucilage obtained as 71.67%. The capsule formulation consisted of a 39% powder of BS mucilage (60 meshes); 50% MCC (60 meshes) as the filler; both 10% CMC-Na and PVPP XL-10 (3:1, 60 meshes) as the disintegrants; both 1% colloidal silicon dioxide and sodium stearyl fumarate (1:1, 100 meshes) as the glidant; by weight, for the preparation of a 320 mg/grain capsule with a 154.7 ± 0.95 mg/g polysaccharide content. Our findings provide a reference for the development and utilization of BS mucilage in healthcare products, which are suitable for the production of bulk samples.

## Figures and Tables

**Table 1:** A CCD matrix of the four variables in coded units and response values for the extraction yield of BS mucilage.

Run	A (°C)	B (mL/g)	C (min)	D (rpm)	Extraction Temperature (°C)	Liquid-Solid Ratio (mL/g)	Extraction Time (min)	Shear Speed (rpm)	Extraction Yield (%)
1	0	0	2	0	80	40	140	8000	54.755
2	1	−1	−1	1	90	30	80	10,000	54.450
3	1	1	−1	1	90	50	80	10,000	56.776
4	0	0	0	−2	80	40	100	4000	59.878
5	2	0	0	0	100	40	100	8000	59.549
6	0	0	0	0	80	40	100	8000	68.409
7	0	0	−2	0	80	40	60	8000	47.141
8	−1	−1	−1	−1	70	30	80	6000	50.878
9	0	0	0	2	80	40	100	12,000	69.490
10	−1	−1	1	1	70	30	120	10,000	52.734
11	−2	0	0	0	60	40	100	8000	52.123
12	1	1	−1	−1	90	50	80	6000	57.552
13	0	2	0	0	80	60	100	8000	61.735
14	−1	−1	1	−1	70	30	120	6000	49.162
15	−1	1	−1	1	70	50	80	10,000	53.040
16	0	0	0	0	80	40	100	8000	69.490
17	−1	1	1	1	70	50	120	10,000	67.469
18	−1	1	1	−1	70	50	120	6000	56.917
19	0	−2	0	0	80	20	100	8000	32.736
20	1	1	1	−1	90	50	120	6000	59.714
21	−1	−1	−1	1	70	30	80	10,000	47.306
22	1	−1	1	−1	90	30	120	6000	45.755
23	1	−1	−1	−1	90	30	80	6000	56.142
24	−1	1	−1	−1	70	50	80	6000	48.387
25	1	−1	1	1	90	30	120	10,000	56.612
26	1	1	1	1	90	50	120	10,000	68.080

**Table 2:** Variance analysis of response surface quadratic model for the extraction of BS mucilage.

Source	Sum of Squares	df	Mean Square	*F* Value	*P*-Value (Prob > F)	Significance
Model	1739.21	14	124.23	13.09	<0.0001	**
A-Temperature	80.81	1	80.81	8.52	0.0140	*
B-Liquid-solid ratio	531.04	1	531.04	55.97	<0.0001	**
C-Time	92.59	1	92.59	9.76	0.0097	**
D-Shear speed	109.16	1	109.16	11.51	0.0060	**
AB	0.74	1	0.74	0.077	0.7859	
AC	28.70	1	28.70	3.03	0.1098	
AD	0.15	1	0.15	0.016	0.9022	
BC	104.74	1	104.74	11.04	0.0068	**
BD	11.61	1	11.61	1.22	0.2922	
CD	75.40	1	75.40	7.95	0.0167	*
A^2^	180.33	1	180.33	19.01	0.0011	**
B^2^	502.27	1	502.27	52.94	<0.0001	**
C^2^	343.50	1	343.50	36.20	<0.0001	**
D^2^	17.53	1	17.53	1.85	0.2013	
Residual	104.37	11	9.49			
Lack of Fit	103.78	10	10.38	17.76	0.1828	Not significant
Pure Error	0.58	1	0.58			
Cor Total	1843.57	25				

** Means significant differences (*P* < 0.01), * Means significant differences (*P* < 0.05).

**Table 3:** The effects of high-temperature, high-humidity and strong light exposure on the weight and polysaccharide content of BS mucilage.

Time (d)	Temperature (60 °C)	Humidity (95% ± 5%)	Illuminance (4500 lx ± 500 lx)
0 (Powder weight)	4.7494 g	4.3586 g	4.3205 g
5 (Powder weight)	4.8335 g	5.6601 g	4.7181 g
10 (Powder weight)	5.0036 g	7.5229 g	5.1489 g
0 (Polysaccharide content)	52.97%	53.05%	53.02%
5 (Polysaccharide content)	52.68%	52.69%	52.79%
10 (Polysaccharide content)	52.54%	52.67%	52.87%

**Table 4:** The screening results of the disintegrants.

Disintegrants	For-1	For-2	For-3	For-4	For-5	For-6	For-7	For-8
Else (mg)	30.4	30.4	30.4	30.4	30.4	30.4	30.4	30.0
CCS (mg)	0.8	—	0.8	—	—	0.8	—	—
CMS-Na (mg)	—	0.8	—	0.8	—	0.8	1.2	1.5
PVPP XL-10 (mg)	0.8	0.8	—	—	0.8	—	0.4	0.5
PVPP XL (mg)	—	—	0.8	0.8	0.8	—	—	—
Disintegration time (min)	>15	11	>15	13	>15	13	10	7

**Table 5:** The screening results of the fillers.

Fillers	For-1	For-2	For-3
MCC (Shanhe) (mg)	160	—	—
MCC (JRS) (mg)	—	160	—
Calcium hydrophosphate (mg)	—	—	160
Disintegration time (min)	26	20	35
Angle of repose (°)	41.5	39.3	42.9

**Table 6:** The screening results of glidants.

Glidants	For-1	For-2	For-3	For-4	For-5	For-6
Colloidal silicon dioxide (mg)	—	1.6	1.28	1.6	1.28	—
Magnesium stearate (mg)	3.2	1.6	1.92	—	—	—
Sodium stearyl fumarate (mg)	—	—	—	1.6	1.92	3.2
Angle of repose (°)	40.1	38.5	39.7	37.8	38.9	39.7

**Table 7:** Evaluation of the capsule by BS mucilage.

Projects	Sample-1	Sample-2	Sample-3	Mean Values
Water content (%)	4.56	5.22	4.67	4.81 ± 0.402
Weight (g)	0.3276	0.3261	0.3282	0.32729 ± 0.007
Polysaccharide content (mg/g)	163.2	144.4	156.5	154.7 ± 0.95
